# Prevalence and characteristics of medication errors at an emergency department of a teaching hospital in Malaysia

**DOI:** 10.1186/s12913-020-4921-4

**Published:** 2020-01-22

**Authors:** Zayyanu Shitu, Myat Moe Thwe Aung, Tuan Hairulnizam Tuan Kamauzaman, Ab Fatah Ab Rahman

**Affiliations:** 1 Hospital Services and Management Board, Ministry of Health, Zamfara State, Gusau, Nigeria; 2 Department of Community Medicine, Faculty of Medicine, Universiti Sultan Zainal Abidin, Kota Campus, 20400 Kuala Terengganu, Malaysia; 3 Department of Emergency Medicine, School of Medical Sciences, Universiti Sains Malaysia, Health Campus, 16150 Kubang Kerian, Kelantan Malaysia; 4 Faculty of Pharmacy, Universiti Sultan Zainal Abidin, Besut Campus, 22200 Besut, Malaysia

**Keywords:** Medication errors, Prevalence, Emergency department

## Abstract

**Background:**

Medication use process in the emergency department (ED) can be challenging and the risk for medication error (ME) to occur is high. In Malaysia, several studies on ME have been conducted in various hospital settings. However, little is known about the prevalence of ME in emergency department (ED) in these hospitals.

The objective of this study was to determine the prevalence and characteristics of ME at an ED of a teaching hospital in Malaysia.

**Methods:**

A cross-sectional study was conducted over the period of 9 weeks in patients who visited the ED of Hospital Universiti Sains Malaysia (HUSM), Kelantan, Malaysia. Data on patient medication orders and demographic information was collected from the doctor’s clerking sheet. Observations were made on nursing activities and these were documented in the data collection form. Other information related to the administration of medications were obtained from the nursing care records.

**Results:**

Observations and data collections were made for 547 patients who fulfilled the study criteria. From these, 311 patient data were randomly selected for analysis. Ninety-five patients had at least one ME. The prevalence of ME was calculated to be 30.5%. The most common types of ME were wrong time error (46.9%), unauthorized drug error (25.4%), omission error (18.5%) and dose error (9.2%). The most frequently drug associated with ME was analgesics. No adverse event was observed.

**Conclusions:**

The prevalence of ME in our ED setting was moderately high. However, the majority of them did not result in any adverse event. Intervention measures are needed to prevent further occurrence.

## Background

Medication error (ME) is briefly defined as any preventable event that may lead to inappropriate medication use or cause patient harm while the medication is in the control of the healthcare professional, patient, or consumer [1]. It is a worldwide issue concerning patient safety and is an important cause of morbidity and mortality [2–4]. In the USA alone, medication errors cause injury to at least 1.5 million people and costing USD3.5 billion to treat annually [5]. Globally, the economic impact of ME is estimated at USD42 billion every year [6]. There has been a rise in global attention on safe medication practices. In March 2017, the WHO launched the “Medication without Harm”, which is a global patient safety initiative aiming to reduce severe, avoidable harm related to medications in all countries by 50% in the next 5 years [6].

Hospital-based studies on ME have been conducted in different settings. These settings involved outpatients [7–9] and inpatients departments of hospitals [10–12]. In addition, there are also studies conducted in the emergency departments [13, 14]. The emergency department (ED) is busy in nature and has a heavy workload with multiple specialties, disease conditions and medications [13, 15–18]. These conditions make the ED setting more medication error-prone.

Data on medication error in the southeast Asia region is lacking [4]. In Malaysia, several studies have been conducted in inpatient settings [19–21] as well as in outpatient pharmacy [22]. The rates of ME reported from these studies varied between 11.7 to 97.7%. To our knowledge there has been no study in Malaysia providing an insight into the occurrence of ME in an ED setting. Since ME can occur at various stages of a medication use process, in this study we chose to explore errors associated with drug administration. Therefore, the objectives of this study were to determine the prevalence and describe the characteristics of drug administration errors in the emergency department of a teaching hospital in Malaysia.

## Methods

### Study design and setting

A cross-sectional study was conducted from October 2017 to December 2017 in the Emergency Department of the Hospital Universiti Sains Malaysia (HUSM). HUSM is located in the state of Kelantan, Malaysia. It is a 767-bed tertiary hospital with about 65,000 patients attending the emergency department (ED) annually [23]. It is considered the largest referral center in the east coast of Malaysia [24]. As with other emergency departments in the country, the HUSM ED follows the triage system and medical care is provided in separate treatment areas [25]. They are the red (i.e. for critical cases), yellow (i.e. semi critical cases), and green zones (i.e. less or non-critical cases). An observation ward is situated within the vicinity of the HUSM ED.

### Study participants

The nurses and doctors who worked in the ED were invited to participate in the study. They were given a briefing about purpose and nature of the study. All nurses and doctors consented to participate. The study was carried out among adult patients of 18 years old and above, who visited the ED from 8 am to 5 pm; Sundays to Thursdays (i.e. working days in Kelantan). The patients in the observation ward, in the resuscitation section of the red zone, and those who were not prescribed any medication were excluded from the study.

### Data collection procedures

Once the patient was selected, one of the investigators (ZS) prospectively observed and recorded the process of medication administration by the nurses. To determine each incidence of omission error, wrong time error, dose error, unauthorized error, and dosage form error, data in the medical record was compared with the data in the respective nursing medical chart. Wrong technique and deteriorated drug errors were determined from direct observation. As for route of administration error, it was determined from both direct observation and comparison of data documented in medical record and nursing medication chart. These processes were done in the red, yellow, and green zone consecutively. For each day, data were collected from all patients who met the study criteria in only one treatment zone. Then, the required samples were selected randomly from the list of the sampling frame. We tried to minimize Hawthorne effect by conducting a trial period of 2 weeks before the actual data collection was made.

In this study, we excluded prescribing errors. Only drug administration errors were included. The definitions for types of administration error were adopted from the literature [19, 26–29]. A medication error was considered as an omission error when the prescribed medication was not administered to the patient before the next scheduled medication or was not administered at all. Medication administered to the patients 1 h before or after the scheduled prescribed time was considered as wrong time error. Unauthorized error occurred when a medication that was not prescribed by the doctor was administered. Dose error was considered when the medication administered to the patient has a greater or lesser strength than that prescribed by the doctor. Medications administered by a different route other than that prescribed by the doctor was regarded as route of administration error. Inappropriate procedure or improper technique in the administration of a medication was regarded as wrong technique error. Dispensing or administration of a medication that has expired, or where its physical or chemical dosage-form integrity has been compromised was regarded as deteriorated drug error. Administration of a medication with a dosage form other than that prescribed was considered as dosage form error.

Demographic data of patient and medications prescribed by the doctor were obtained from each patient medical record. Data on medications, times medication administered, doses, routes, and the person(s) who administered the medications were obtained from the nursing medication chart.

### Sample size calculation and sampling

A single proportion formula is a formula used to determine the ratio in the total population from which our sample were drawn from. This was applied in this study because we have access to the total patient population of patients visit to the ED during our study period, and our sample was drawn from the total visit. The number of subjects required was calculated using the single proportion formula [30], assuming 75.1% as the expected proportion [31] and 0.05 significance (alpha) level at 95% confidence interval (CI). The final sample size was determined to be 311. Simple random sampling method was applied to select the required samples.

### Data analysis

Data were analysed using Statistical Package for Social Sciences software (SPSS version 22, IBM Corp., USA). The prevalence of medication error was determined by dividing the number of patients with ME by the total sample size. Descriptive statistics was applied, and results were presented as frequency (%) for categorical data and mean (standard deviation) for numerical data to describe the characteristics of those patients with ME.

### Ethical approval

This study was approved by the Human Research Ethics Committee (HREC) of Universiti Sains Malaysia (USM/JEPeM/17050250).

## Results

There were 10,874 ED visits over the nine-week period. A total of 5454 patients visited the ED between 08:00 am to 05:00 pm. Observations and data collections were made for 547 patients who fulfilled the study criteria. From these, 311 patient data were randomly selected for analysis.

There were more male (53.7%) than female patients (46.3%). Their mean age (SD) was 44.02 (18.39) years old with the minimum age of 18 years old and the maximum age of 84 years old (Table 1). There was a higher number of patient-visit in the late afternoon session compared to the morning and early afternoon. Various medical diagnoses were reported during the ED visit and the mean number of medications received by the patients was 2.68 (range: 1 to 12). Table 2 shows number of medication administrations by treatment zones.

**Table 1:** Demographic profile and clinical characteristics of patients (*n* = 311)

Variables	Frequency (%)	Mean (SD)	*P*-value^*^
Gender			
Male	167 (53.7)		0.192
Female	144 (46.3)		
Age (years)		44.02(18.39)	
18–25	77 (24.8)		< 0.001
26–40	60 (19.3)		
41–60	113 (36.3)		
60+	61 (19.6)		
Ethnicity			
Malay	282 (90.7)		< 0.001
Chinese	12 (3.9)		
Indian	17 (5.5)		
Time of visit			
Morning session (8 am-12 pm)	108 (34.7)		0.301
Early afternoon session (12 pm–2 pm)	91 (29.3)		
Late afternoon session (2 pm–5 pm)	112 (36.0)		
Triage			
Red	84 (27.0)		0.004
Yellow	131 (42.1)		
Green	96 (30.9)		
Having allergic history			
No	278 (89.4)		< 0.001
Yes	33 (10.6)		
Concurrent medical problem			
No	242 (77.8)		< 0.001
Yes	69 (22.2)		
Concurrent medication			
No	245 (78.8)		< 0.001
Yes	66 (21.2)		
Medical diagnosis classification^**^			
Diseases of the circulatory system	69 (22.1)		
Diseases of the respiratory system	62 (19.9)		
Injury, Poison and certain other consequences of external cause	56 (18.0)		
Diseases of the digestive system	25 (8.0)		
Endocrine, nutritional or metabolic diseases	20 (6.4)		
Diseases of the skin	16 (5.2)		
Neoplasm	15 (4.8)		
Disease of genitourinary system	14 (4.5)		
Diseases of the blood or blood-formingorgans	9 (2.9)		
External causes of morbidity or mortality	9 (2.9)		
Disease of the musculoskeletal system or connective tissue	6 (1.9)		
Certain infectious or parasitic diseases	4 (1.3)		
Diseases of the nervous system	3 (1.0)		
Symptoms, signs and clinical findings not elsewhere classified	3 (1.0)		

^*^Chi-square goodness-of-fit; ^**^Classification based on ICD-11

**Table 2:** Drug administrations by treatment zone

Treatment zones	No. of patients	No. of medications	Mean (SD)	*F* statistics (*df*)	*P* value^*^
Red zone	84	362	4.31 (2.53)		
Yellow zone	130	272	2.09 (1.18)	57.95 (2, 308)	*P* < 0.001
Green zone	97	201	2.07 (1.01)		

^*^One-way ANOVA

Post hoc analysis: The mean number of drug administrations were significantly different between the red zone and the yellow zone (*p* < 0.001) as well as between the red zone and the green zone (*p* < 0.001). However, there was no significant difference of mean number of drug administrations between the yellow zone and the green zone (*p* > 0.950)

A total of 130 medication errors were detected in 95 patients. The prevalence of medication errors was found to be 30.5% (95% CI of percentage: 25.3, 35.7). The highest number of medication error was detected among patients getting treatment in the red zone (56.8%), followed by the yellow zone (35.8%) and the green zone (7.4%). There were more male patients than female patients involved (60 vs 35).

The most commonly diagnosed medical problems among these patients were related to cardiovascular and respiratory diseases. Various medications were administered, the most common being analgesics (42.1%), cardiovascular drugs (43.2%), antibacterial (29.5%) and gastrointestinal drugs (21.1%). Table 3 shows medications that were implicated in ME according to types of ME.

**Table 3:** Medications implicated with Medication Error (*N* = 95)

Class of medications prescribed	N (%)
Analgesics	47 (49.40)
Cardiovascular medications	34 (35.70)
Antiinfectives	28 (29.40)
Gastrointestinal medications	20 (21.05)
Central nervous system medications	12 (12.63)
Anti-inflammatory/antirheumatic/gout	7 (7.36)
Vaccines, antisera and immunological	5 (5.26)
Respiratory medications	5 (5.26)
Anesthesia and antimuscaric agents	4 (4.21)
Endocrine products and sex hormones	2 (2.10)

Four types of medication errors were detected in these patients. Table 4 shows the distribution of the types of medication errors. The commonest type of medication error was wrong time error, which occurred in 61 cases (46.9%). There was no adverse event observed due to medication error during this study period.

**Table 4:** Types of medication errors (130 ME in 95 patients)

ME type	N	%
Wrong time	61	46.9
Unauthorised	33	25.4
Omission	24	18.5
Dose	12	9.2
	130	100%

% based on total number of ME

## Discussion

The prevalence of medication error in the emergency department reported by previous studies vary widely between 20 to 70% [32–36]. This wide variation in prevalence has been attributed to differences in definitions, study design, patient selection and other factors. Some studies reported the prevalence of ME was based the total drug administrations [35] while others reported prevalence based on the number of patients [32–34, 36]. Prospective study designs where incidences are actively being solicited, tend to report higher prevalence [34, 35]. Patient selection can also influence study findings. Marcin et al. [33] reported a prevalence of about 40% among children attending the emergency department. The relatively high prevalence could be attributed to their patient selection where only the sickest children were selected for the study.

Among the types of medication errors identified in this study, wrong time error was the most prevalent, followed by unauthorised drug error and omission error. In the ED, patients are constantly subjected to various medical and diagnostic procedures. These procedures could sometimes take more time than expected and would interfere with the scheduling of the medication. On the other hand, patients in the hospital ward are usually on a regular schedule of medications. Thus, a relatively lower rate of wrong time error would be expected in inpatient settings [20, 31, 37]. Unauthorized drug error was the second most prevalent type of medication error observed in this study. This type of error could be due to medication brand confusion and verbal orders of drugs [38], or misreading the medical file by the nurses [19]. In our setting, most of the medications involved in unauthorized drug error were medicines given by nurses on standing orders by the doctors. Although standing orders are widely practiced by emergency department doctors and nurses, currently, it is still an undocumented policy in the ED of our hospital.

The prevalence of omission error in our study was somewhat similar than what has been determined in previous studies [13, 35, 36]. A higher prevalence of 77.6% has been reported by Acheampong et al. [39], probably due to the inclusion of “unavailability of medicine” in their definition of omission error. Omission errors could be due to lack of documentation of medication process on the nurse’s treatment sheet when changing from one shift to another. This lead to some of the scheduled medications not being administered. Dose error was the least prevalent type of medication error identified in this study. The prevalence was relatively lower compared with the findings from emergency departments elsewhere [13, 36]. Dose error is mostly caused by the wrong transcription by doctors and nurses, poor handwriting, and poor drug preparation skills and knowledge by the nurses [21, 40]. Due to the nature of the handwritten prescriptions by the doctors, sometimes it could be hard to interpret the order.

A variety of measures have been recommended to minimise the incidence of medication error. Wrong time error can be minimized through the use of electronic medication administration record (EMAR). EMAR works by automatically tracking medications from order to administration. Since the system uses barcode scanning technology, it reduces documentation time as well as medication errors. Unauthorized drug error can be categorized as a rule-based error. Rule-based errors are failures to apply the good rule or the application of the bad rule in a setting. This includes the non-documented standing orders given in the emergency department causing unauthorized medication errors. This cause of error can be minimized through the use of standards and good rules of practice in a hospital setting [41]. Omission errors are the most difficult type of error to prevent from happening. The memory-based error can be minimized through putting in place systems that can check such errors such as computerization and checklists that could be used as remedies to the action. To avoid poor documentation, bar-coded assisted medication administration record can be used. Marx et al. [42] and Helmons et al. [43] showed how barcode results in a positive outcome, in which the proportions of MEs were reduced by 50 and 58%, respectively. In addition, nurses must generate the missed medications and give medication report on the treatment sheet at the end of every shift. It will help in confirming to all nurses that they have completed their scheduled medication administrations. A warning or reminder must be in place if any medication is missed to avoid omitting it by the next batch of nurses in a subsequent work shift. The absence of standardized handoff communication has been highlighted as one of the barriers to achieving medication-safety-related National Patient Safety Goals [44]. Dose error is regarded as an action-based error and can be minimized through the use of technological interventions. Improving the nature of prescription through the use of computerized physician order entry (CPOE) could reduce the rate of dose error. Previous studies showed CPOE resulted in a reduction in the rate of prescribing error and improved patient safety and cost saving [45, 46].

During the period of this study, no adverse event due to medication error was observed. In Malaysia, a recent study on medication errors reported to the National Medication Error Reporting System showed that 98% of the cases did not result in harmful effects [47]. Similarly, other studies [48, 49] have reported that the majority of medication errors did not result in any harm to the patients.

### Implication and future directions

In this study we excluded prescribing errors although this type of error constituted the majority of ME reported [50]. However, prescribing errors are more likely to be intercepted compared to drug administration errors, the latter being more likely to reach the patients [51, 52].

This is the first study looking at drug administration errors in an ED in Malaysia. Admissions through the ED of public hospitals throughout the country has been increasing every year. For example, admissions to the ED in 2009 was 6,745,721 [53] and by 2016 it has increased to 8,253,303 admissions, an increase of more than 20% [54]. The ED is busy in nature and has a heavy workload with a greater heterogeneity of patients and medications. These conditions increase the potential for compromised patient safety in the ED. At the same time, nationwide data shows that reporting of ME has been steadily increasing every year. In 2009 there were 2626 MEs reported to the national center [47]. By 2017 the center received almost 20,000 ME reports [50].

Our findings show that the types of ME (i.e. wrong time, unauthorised drug, omission, and dose errors) that occurred in our setting underscore the importance of documentation and real-time monitoring. Although some hospitals in the country have implemented the Total Hospital Information System (THIS) [55], paper-based medical records are the way most hospitals continue to preserve their patient medical records. Only 21 out of 138 public hospitals have been implementing various forms of hospital information systems [56]. However, recently, the Minister of Health announced that Malaysia is planning to implement electronic medical record with 5G technology by mid 2020 [57].

There are potential benefits associated with THIS but some of the barriers and challenges must be addressed [55, 58, 59]. Financial commitment to install and maintain such a system is enormous. The success in the implementation of such system was usually due to staff commitment to the system and ongoing training [60]. Further studies need to find out how this implementation will impact existing workflow processes and patient safety. Feasibility studies to implement the system should take into account these barriers.

### Limitations of the study

The pattern of ED visits from our study resembled what has been reported previously at the same hospital [61]. Selasawati et al. [61] found that more patients visited the ED from 6 pm to 10 pm and during the weekends (Friday and Saturday). However, in our study we have excluded those patients who came to the ED during these time periods due to lack of manpower; only one person was involved in collecting the actual data. Since negative patient outcomes increase with increased workload [62], more occurrence of ME would be expected to occur at night than during the daytime. Higher medication errors at night have been related to less supervision and the onset of fatigue among physicians during the night [63]. Therefore, the currently estimated prevalence obtained in our study could have been an underestimation of the overall prevalence of ME in this setting.

## Conclusion

The prevalence of medication error in the ED at a tertiary hospital in Malaysia was moderately high but did not result in any adverse event. The occurrence of medication errors in our setting could be avoided or reduced with appropriate measures.

## Data Availability

The datasets generated and/or analysed during the current study are not publicly available due to data confidentiality policy per ethical approval but are available from the corresponding author on reasonable request.

## Abbreviations

CPOE: Computerized Physician Order Entry
ED: Emergency Department
HUSM: Hospital Universiti Sains Malaysia
ME: Medication error
USA: United States of America
WHO: World Health Organization
